# Influence of Ulluco Starch Concentration on the Physicochemical Properties of Starch–Chitosan Biocomposite Films

**DOI:** 10.3390/polym13234232

**Published:** 2021-12-02

**Authors:** Luis Daniel Daza, Valeria Soledad Eim, Henry Alexander Váquiro

**Affiliations:** 1 Departamento de Química, Universidad de las Islas Baleares, 07122 Palma de Mallorca, Spain; danieldaza08@gmail.com; 2 Departamento de Producción y Sanidad Vegetal, Facultad Ingeniería Agronómica, Universidad del Tolima, Ibagué 730006, Colombia

**Keywords:** packaging, chitosan, biopolymer, films, *Ullucus tuberosus* Caldas

## Abstract

This work aimed to prepare ulluco starch (US)/chitosan (Ch) edible films and evaluate the effect of the concentration of US on their physicochemical properties. The use of edible films is a means of adding value to the ulluco crop and evaluating the viability of using new sources to produce packaging materials. Different samples were prepared at different US concentrations (2%, 3%, 4%, and 5% *w*/*v*) and a fixed chitosan concentration (1.5% *w*/*v*); then, samples were analyzed, considering their physical, mechanical, and thermal properties. The US/Ch edible films showed an increase in solubility from 17.5% to 21.7%, swelling power (SP) from 38.9% to 267%, tensile strength (TS) from 3.69 MPa to 10.7 MPa, Young modulus (YM) from 18.0 Pa to 652 Pa, and thermal stability as the US concentration increased. However, samples with low US concentrations showed higher elongation at break (EB) (36.6%) and better barrier properties (WVP) (5.61 × 10^−11^ g/m s Pa). The films evaluated in this work presented good physical, mechanical, and barrier properties, revealing their potential as packaging material ensuring food security, and demonstrating the technological potential of US.

## 1. Introduction

One of the main strategies of the food industry for product preservation, conservation, storage, and transportation is the use of packaging. Over the last 50 years, conventional synthetic or petroleum-based plastic packaging such as polypropylene (PP), polystyrene (PS), and polyethylene terephthalate (PET) has adequately accomplished this task [1,2]. However, there are now new concerns regarding the use of these types of plastic, such as the fact that they are single-use, which, added to their low biodegradability, can generate pollution [3]. Their impact on the environment has been such that different policies have been implemented worldwide to reduce the use of single-use plastics [4]. These policies create significant challenges for the industry, and the development and design of eco-friendly packaging is one of the alternatives most explored by researchers [5]. Different investigations have been conducted to evaluate film-forming materials and to study the effect on the physicochemical properties of these films, in order to obtain materials that have similar characteristics to the existing petroleum-based plastics and also provide additional benefits such as antimicrobial and antioxidant properties [6]. However, the huge demand for packaging for the industry requires the search for new sources of materials for the development of this biodegradable packaging; sources which must have certain characteristics such as biodegradability, non-toxicity, biocompatibility, film forming properties, and low cost. Starch is the main carbohydrate reserve in plants and consists of amylose composed of α-(1,4)-D-glucopyranose subunits and amylopectin composed of α-(1,4)-D-glucan short chains linked through α-1,6 linkages. In addition, starch is a renewable, low-cost, and biodegradable material, which makes it a raw material with high potential in the packaging industry.

*Ullucus tuberosus* Caldas, commonly known as ulluco, olluco, chugua, or papalisa, is an Andean tuber belonging to the Basellaceae family cultivated at high altitudes from Bolivia to Ecuador and Colombia [7]. This crop presents a high starch content, which displays good film-forming properties due to the high amylose content [8,9]. Previous studies have reported the physicochemical properties of ulluco starch (US) and the obtaining of edible films based on US, which presented good mechanical properties, low opacity, and intermediate solubility and swelling properties, indicating its potential as a packaging material [10,11]. On the other hand, these edible films showed high water vapor permeability (WVP) and low antimicrobial capacity, highlighting the need to look for alternatives to improve the barrier and active properties of US-based films. The addition of different active ingredients such as essential oils [12], natural extracts [5], or the mixture with other polymers such as gelatin [13], gums [14] or chitosan [15] are strategies to overcome these problems and have been successfully tested.

Chitosan (Ch) is a linear polycationic amino-polysaccharide extracted from crustaceans or mushrooms, consisting of (1,4)-linked-2-amino-deoxy-β-d-glucan obtained by the partial deacetylation of chitin [16,17]. It is considered a biodegradable, non-toxic, renewable, and biocompatible material, which shows antimicrobial capacity [18]. Some studies have demonstrated the compatibility of starch and Ch for the preparation of film, and the improvement in their mechanical and active properties [15,19]. Currently, there is no evidence of the use of US blended with Ch for the preparation of edible films, which is due to the low exploitation of ulluco as a raw material for industrial processes. Although the use of other starches with chitosan for the preparation of biodegradable films has been evaluated, the interaction of each of these materials is different, which is due to factors such as amylose content, starch purity, and structural configuration of the granule, among others. It is therefore necessary to evaluate the behavior of new starches with chitosan and thus be able to establish the potential of the mixture of these materials for use as packaging.

In this sense, as an alternative to non-biodegradable packaging, this work aims to prepare edible films based on US mixed with Ch and evaluate the effect of starch concentration on their physicochemical and thermal properties. This work will contribute to understanding the properties and US potential as a raw material for the industry.

## 2. Materials and Methods

### 2.1. Materials

Ch (Low molecular weight 50–190 kDa, Deacetylated 75–85%) was purchased from Sigma-Aldrich (Sigma Aldrich, St. Louis, MO, USA), US (amylose content: 35.3%) was previously obtained as reported by Galindez et al. [10]. Food grade acetic acid was purchased from Cimpa S.A.S (Cimpa S.A.S., Bogotá, Colombia), glycerol, and Tween^®^ 80 (Density at 20 °C, 1060 kg m^−3^) were purchased from Merck (Merck, Darmstadt, Germany).

### 2.2. Edible Films Preparation

Ch solution: Ch was dissolved in 1% *v/v* acetic acid under agitation for 1 h at 25 °C to obtain a final solution of 1.5%. The chitosonium acetate solution was allowed to stand overnight and stored at 4 °C [20].

US solution: US was dissolved in distilled water at different concentrations 2%, 3%, 4% and 5% (*w/v*). US mixtures were heated to 95 °C and maintained at this temperature for 5 min. Then, the mixtures were cooled to 60 °C.

For edible film preparation, the Ch solution was heated to 60 °C and mixed with each US solution in a 1:1 (*v/v*) ratio. The mixtures were stirred, and then glycerol (1% *w/v*) and Tween^®^ 80 (0.1% *v/v*) were added (Table 1). After 10 min of agitation, the solution was passed through a peristaltic pump (to eliminate the bubbles). The prepared solutions (30 g) were poured into Petri dishes (Diameter: 8.5 cm) and dried at 40 °C for 24 h using a forced air-flow oven. Before the analysis, the samples were stabilized in a desiccator cabinet (DH-1002, Acequilab, Bogotá, Colombia) at a relative humidity (RH) of 54% and a room temperature (~20 °C) for at least 24 h.

### 2.3. Moisture Content, Swelling Power, and Solubility

Moisture content (MC) g of water/100 g of sample (% w.b.), swelling power (SP, %), and solubility (%) of edible films were determined by following the gravimetric method described by Homez-Jara et al. [20]. Results were reported as the average of five repetitions.

### 2.4. Color and Transparency

The color parameters were assessed using a Minolta colorimeter (Cr 410, Konica Minolta, Tokyo, Japan) according to [11]. Results were expressed as yellow index (YI) and color index (CI). The opacity of the films was assessed following the methodology described by [11]. Measurements were performed on the film samples at least eight times, and the average results were reported.

### 2.5. Water Vapor Permeability

The water vapor permeability (WVP) was evaluated according to the ASTM method [21]. The film samples were loaded into stainless steel permeability cells, in which 6 mL of distilled water was deposited with an exposed area of 9.6 cm^2^. It was assumed that the RH was maintained at 100% within the cell. The cells were placed in a controlled chamber at 25 ± 2 °C and 75 ± 2% RH. Every hour, the mass of the cells was recorded using an analytical balance (±0.0001 g) for 8 h. A fan was placed inside the chamber to ensure uniform conditions within the chamber. The WVP (g m^−1^ s^−1^ Pa^−1^) was calculated using Equation (1):

(1) $$\mathrm{WVP}=aFT/tAP_{sat}\left(RH_{1}-RH_{2}\right)$$

where a is the mass of the permeability cell (g) at time t (s); FT is the film thickness (m); A is the exposed area (m^2^); *P_sat_* is the saturation vapor pressure at the test temperature (Pa); *RH*_1_ is the RH of the water contained in the cells, expressed as a fraction; and *RH*_2_ is the RH of the chamber, expressed as a fraction. Measurements were performed on the film samples at least four times.

### 2.6. Mechanical Properties

The tensile properties of the films were evaluated following the ASTM methodology [22] using a texturometer (LS1, Lloyd Ltd., Largo, FL, USA). The films were cut into 5 cm × 1.0 cm strips, and the crosshead speed and initial grip separation were set to 5 mm/min and 4 cm, respectively. The tensile strength (TS, MPa), Young modulus (YM, MPa), and elongation at break (EB, %) were determined using Nexygen Plus software (Lloyd ltd., Largo, FL, USA, Version 3.0). Results were reported as the average of six measurements.

### 2.7. Morphology of Edible Films

The surface morphology of US and edible films was examined by scanning electron microscopy (SEM; Quanta 650 FEG, FEI Company, Hillsboro, OR, USA). The samples were placed on stubs using double-sided carbon tape and covered with a gold layer using a rotary-pump coating system (Q150R, Quorum, Lewes, UK). Images were obtained at an accelerating voltage of 10 kV and varying degrees of magnification.

### 2.8. X-ray Diffraction of Edible Films

X-ray diffraction (XRD) analysis of the US, Ch, and films was carried out using a Bruker D8 ADVANCE diffractometer (Bruker, Billerica, MA, USA) operating with Ni-filtered Cu-Kα1 radiation at 40 kV and 40 mA and a specific wavelength of 1.5406 Å. Data collection was carried out in the 2θ range of 3.5–70°, with a step size of 0.02035° (2θ) and a counting time of 0.8 s/step. Relative crystallinity was determined using the relationship between the peak area and total area.

### 2.9. Fourier-Transform Infrared (ATR-FTIR) Spectroscopy of Starch and Edible Films

The Fourier-transform infrared spectroscopy (ATR-FTIR) spectra of pure components and edible films were recorded in Fourier transform infrared spectroscopy (FTIR, IRAffinity-1S, Shimadzu, Japan). For the analysis of solid samples, pellets were prepared by a mixture and homogenization of the sample with potassium bromide (KBr), and for liquid samples, a liquid cell was used. The measurements were taken at room temperature, in triplicate, and recorded over the range of 4000–700 cm^−1^, with a resolution of 4 cm^−1^ and 20 scans.

### 2.10. Contact Angle Measurement

The contact angle (θ) of samples was determined by the sessile drop method using an automated drop tensiometer (Tracker S, TRACKER™, Teclis Scientific, France). A droplet of 5 µL of distilled water was deposited on the surface of the films. The contact angle was measured after 5 s and expressed in degrees [23]. The results were expressed as the mean of at least five measurements taken at random positions on the samples.

### 2.11. Thermal Analysis

Differential scanning calorimetry (DSC) analysis was performed using a differential scanning calorimeter (DSC 3+, star system, Mettler Toledo, Columbus, OH, USA). Approximately 3 to 6 mg of the samples were placed into aluminum pans and hermetically sealed. The pans containing the samples were cooled to −60 °C at 20 °C/min and then heated from −60 °C to 250 °C at a constant rate of 20 °C/min. The whole process was carried out in a nitrogen atmosphere. An empty pan was used as a reference. The obtained thermograms were examined using Universal Analysis 2000 V3.9A software (TA Instruments, Tokio, Japan). The melting temperature (*T*_m_) of each sample was determined. Thermal analyses were carried out in triplicate.

Thermogravimetric analysis (TGA) was performed according to Galindez et al., [10] using a thermogravimetric analyzer (SDT 2960, TA instruments, Tokio, Japan). The weight loss, thermal degradation temperature (*T*_max_), onset temperature (*T*_onset_), and total loss of pure components and edible films were determined over the temperature range of 20–600 °C at a heating rate of 20 °C/min and under a stream of nitrogen. Thermal analyses were carried out in duplicate.

### 2.12. Statistical Analysis

Analysis of variance (ANOVA) at a 95% confidence level was performed to determine significant differences in the properties between samples and storage time. Multiple range tests (MRTs), using the Tukey–Kramer test, were used to determine significant differences between groups. Statistical analysis was performed using the MATLAB^®^ R2017b software (The MathWorks Inc., Natick, MA, USA).

## 3. Results

### 3.1. Moisture Content, Swelling Power, and Solubility

The MC of US/Ch biodegradable films ranged from 15.2% to 33.4% (Table 2). Results showed an inverse correlation between the starch concentration and the MC. Samples S2 and S5 showed the highest and the lowest MC (*p* < 0.05), respectively. This behavior can be explained since a higher concentration of starch is related to a greater amount of hydrophilic groups, which can bind more strongly the water molecules and thus prevent their evaporation. In contrast, its hydrophilic groups interact rapidly with the hydrophilic groups of chitosan and glycerol at low starch concentrations. These will reduce the hydrophilic groups available to interact with the water molecules through hydrogen bonds, leading to a greater amount of free water molecules and consequently greater evaporation and mass loss [20].

The solubility of the samples analyzed increased with a rise in the concentration of US (Table 2), indicating that films with a higher content of US showed greater affinity for water molecules or low water resistance. The solubility values ranged between 17.5% and 21.7%, the highest solubility being shown by S5, followed by S4, S3, and S2 (*p* < 0.05). A possible explanation for this behavior could be that the interactions between the components of the films do not generate steric hindrance due to the formation of a disorganized network gel structure, allowing the entry of water. In addition, the low moisture content of the films with a higher concentration of US increased the susceptibility to interact with molecules of water through hydrogen bonds. The increase in solubility showed similar behavior to the films prepared from gelatin and casein phosphopeptides [24]. In addition, our results are similar to those reported for solubility of films obtained using corn starch and Ch [25] but differ from the high solubility reported for pea starch and Ch [26]. These differences can be attributed to the botanical source of starch, amylose content, the final concentration of the polymers in the film-forming solution, and the molecular weight of the Ch. In our case, the use of Ch with a low molecular weight can contribute to a more significant interaction between polymers.

Moreover, results obtained in this work agree with those reported previously for ulluco edible films [11], indicating that the chitosan did not affect the films’ affinity towards water. The solubility of materials can help determine their applicability to foods. In some applications, such as the coating of products with high moisture content, it is necessary to use materials with low solubility to guarantee their integrity. On the other hand, the coating of products with low moisture content, such as candies, requires materials with high solubility [27].

The SP showed a similar behavior to solubility; as the concentration of starch increased, the ability of the biodegradable films to swell significantly increased (*p* < 0.05) (Table 2). This behavior is characterized by more functional groups of a hydrophilic nature that can interact with water molecules; thus, the higher the US concentration, the greater the SP. Furthermore, as explained above, the degree of disorder of the gel network structure and the lack of steric hindrance increases the interaction capacity between the constituents (US, Ch, and glycerol) of the biodegradable films and the water. In addition, glycerol and chitosan can interact with starch molecules, enhancing free volume in the film network [28]. Similar behavior was reported for films produced using potato starch and Ch [29]. The SP of edible films evaluated in this study was higher than that reported for edible films obtained with starch from the same species and dried at different temperatures [10,11]. The differences observed between films obtained with the same type of polymer are mainly due to the concentration of starch. US edible films showed similar swelling capacities to other films obtained with lentil (126 to 133%) and quinoa starch (255 to 297%), but lower than those obtained using pumpkin starch (387 to 500%) [28].

### 3.2. Water Vapor Permeability

A key characteristic of food packaging is to act as a barrier that prevents the exchange of water and gases between the food matrix and the surroundings, thus delaying the deterioration of the product. The WVP values of US/Ch biodegradable films are shown in Table 2. In general, an increase in the WVP of the samples was observed with an increase of US concentration, which is related to the higher solubility and SP of films with higher US content. However, samples S3 and S4 did not differ in their WVP values (*p* > 0.05). The results obtained in this work were better than those obtained for tapioca starch [30] and the US films [11]. In addition, the results obtained in this work were better than those reported for corn starch and Ch with low or medium molecular weight, but similar to the films prepared using Ch with high molecular weight [31]. Considering that Ch with low molecular weight was used in this work, the observed differences may be due to the amylose content of starches, which is higher in the US (35.3%) than in corn starch (25%). The low WVP of US/Ch biodegradable films indicates its great potential for extending the shelf life of foods. The differences in WVP between pure starch edible films and UC/Ch films may be due to the addition of Ch to the blend, which displayed hydrophobic characteristics.

According to Minh and Yoksan [32], the addition of chitosan to the mixture with starch can decrease the WVP values due to two factors: a) the presence of acetyl groups at the C2 position of the chitosan pyranose ring provides less hydrophilicity to chitosan as compared with the existence of OH− groups of starch; and b) β-D-(1-4) glucopyranosyl linkages of chitosan give it better-packing structure or higher crystallinity than α-D-(1-4) glucopyranosyl linkages of starch, due to the stronger hydrogen bond formation, which leads to less moisture/water absorption and more difficulty in hydrolysis. Since the US concentration in film S5 was higher than in the others, the amount of available hydrophilic groups was greater, which increased the possibility of interacting with water molecules, allowing the migration of water vapor through the films. Different works have shown that the decrease in the starch / Ch ratio has reduced the WVP of the films [27].

### 3.3. Contact Angle

The θ is related to the hydrophilicity or hydrophobicity degree of the edible film surface [33]. The θ of the samples were 31.39° ± 2.17, 62.54° ± 5.58, 56.03° ± 4.98, and 63.22° ± 2.11 for S2, S3, S4, and S5, respectively. According to Vogler [34], US/Ch edible films can be classified as hydrophilic surfaces (θ < 65°). The lowest θ was shown by S2, indicating the highest hydrophilicity (*p* < 0.05). An increase in US concentration greater than 3% in the edible film preparation leads to an increase in the θ, which means a decrease in the hydrophilicity of the samples. The increase in the θ in the S3, S4, and S5 may be related to the fact that an increase in the concentration of US generated an increase in intermolecular interactions, reducing the availability of hydroxyl groups and therefore the interaction of polymers with water molecules [35]. A similar explanation was pointed out for the θ shown by films prepared using native cassava starch and Ch with high molecular weight [32]. In addition, the US retrogradation after the heat treatment to generate the films can help to reduce the hydrophilicity of the samples [36]. It is important to note that the film with the highest θ shows the highest WVP value. Although sample S5 presents lower hydrophilicity than S2, the number of hydroxyl groups available in S2 is greater. Therefore, these can bind water molecules and entrap them in the structure, preventing its passage (lower WVP value). The opposite occurs with S5.

### 3.4. Optical Properties

Color and transparency are important factors when evaluating packaging, as they will influence consumer perception and acceptability of the product. In addition, the optical properties will serve as selection criteria in defining the end use of the food packaging evaluated [37]. The opacity of the US/Ch edible films ranged between 0.31 and 0.40 (Table 2). It was observed that a decrease in the starch concentration or the starch/chitosan ratio yielded films with higher opacity values, which means that S5 showed the highest transparency among the evaluated samples. The results obtained in this work showed that the opacity of the films is related to the MC, which was similar to the behavior reported for edible films obtained from yuba protein [38]. This phenomenon can be related to the intermolecular agglomeration produced by the MC increase, rising the turbidity, and, therefore, the light scattering of films [38]. The behavior of the opacity from US/Ch was contradictory to the results reported for corn starch and Ch films [27]. The differences observed can be attributed to the deacetylation degree of the Ch used in the film’s preparation, the amylose content of the starch or the thickness of films obtained.

YI values are presented in Table 2. S2 showed the highest YI, followed by S3, S4, and S5 (*p* < 0.05). An increase in the US:Ch ratio was characterized by a decrease in the YI of the films, which is associated with the addition of the Ch, and the development of a yellowish coloration due to the presence of β-1-4 linked 2-amino-2-deoxy-D-glucopyranose repeat units [39]; thus, higher US concentration limited the development of this coloration. Similar behavior was reported for the YI of edible films of Ch and tapioca starch [40]. The values of WI ranged between 87.3 and 87.8. As expected, low US:Ch ratios generated films with low WI, resulting from the development of the yellow color mentioned above. However, no statistical differences were observed between the WI of the treatments with an increase in the concentration of starch.

### 3.5. Mechanical Properties

**Figure 1:**
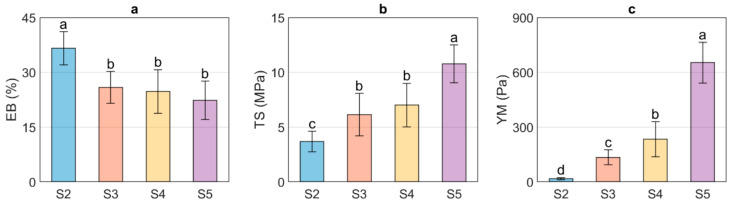
Mechanical properties of the ulluco starch–chitosan edible films. Elongation at break (EB, %) (**a**), tensile strength (TS, MPa) (**b**), and Young Modulus (YM, Pa) (**c**). Different letters indicate significant differences between samples (*p* ≤ 0.05).

Figure 1 shows the EB, TS, and YM values obtained for US/Ch edible films. It was observed that the EB of the evaluated samples decreased as the US concentration increased; however, no differences were observed between samples S3, S4, and S5. The decrease of the EB with an increase in US can be related to the MC since water molecules can act as a plasticizer and thus improve the flexibility of the edible films. Therefore, adding greater amounts of starch will reduce the EB due to the interactions between starch and water molecules [41,42]. In contrast, the samples with the highest values of MC show the lowest values of TS, indicating that the higher the US concentration, the higher the TS of the films. The above is associated with an increase in the number of hydrogen bond interactions between the NH3+ groups, formed from protonation in an acid medium (acetic acid solution) of the amino groups of chitosan backbone, and the OH− of the US exposed by the destruction of the ordered crystalline structure in the gelatinization process [27]. YM is related to the stiffness of a material, which means that materials with high values of this parameter will be rigid. In contrast, materials with low YM values will present more flexible characteristics. As expected, the YM of the US/Ch edible films increased at higher US concentration, which can be explained due to the interaction between the NH3+ and the OH− groups mentioned above. US/Ch films showed higher EB, TS, and YM than those films obtained using starch from brown rice and Ch [15]. In addition, US/Ch films were more elastic than the films obtained using pea starch and Ch, but in contrast, it presented lower values of YM and TS [26].

### 3.6. Morphology of Edible Films

**Figure 2:**
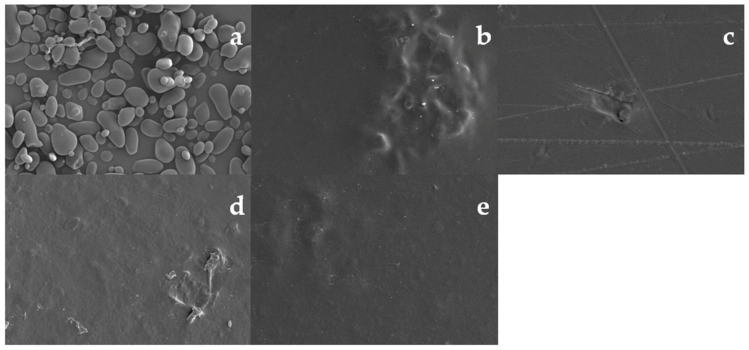
Micrographs of ulluco starch at magnification of 500× (**a**), and micrographs of surface from ulluco starch–chitosan edible films samples: S2 (**b**), S3 (**c**), S4 (**d**) and S5 (**e**) at magnification of 200×.

SEM micrographs of US granules and edible films are shown in Figure 2, where the US granules showed irregular shapes and sizes. However, most granules are characterized by an elongated and rounded shape, with a smooth surface and without evidence of pores or fissures (Figure 2a). The morphology of the US analyzed in this work was similar to that reported for the same species harvested in Argentina [8]. Edible films presented continuous surfaces with no fractures or porosities, implying that the materials were homogeneously mixed. The crowds observed in the figures may be related to problems in the serving process in the Petri dishes. The homogeneity of the mixture of starch and Ch and its effect on the morphology of edible films have been previously reported for pea [41] and corn starches [27].

### 3.7. X-ray Diffraction

**Figure 3:**
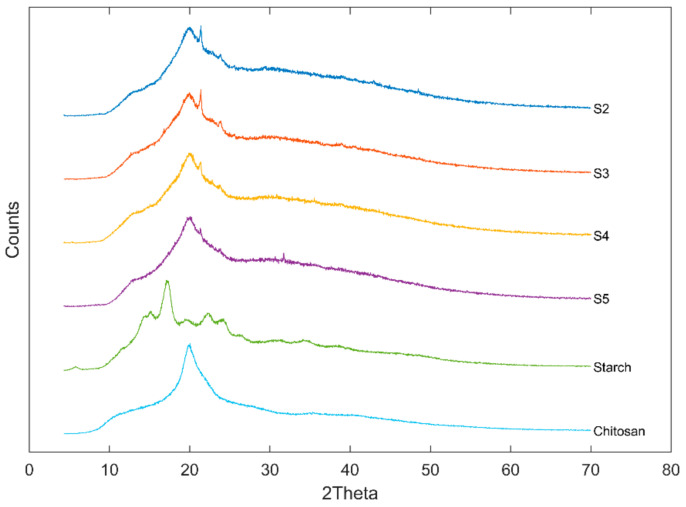
XRD diffractograms of ulluco starch, chitosan, and ulluco starch–chitosan edible films.

Figure 3 presented the XRD patterns of US/Ch edible films, Ch and US. A typical B-type starch diffraction pattern is shown by the native US used in the preparation of the films, which presented peaks at 5.6°, 15°, 17°, 20°, 22° and 24° 2θ. This type of starch is obtained commonly by extraction from tubers [27]. Ch presented a characteristic peak at about 21° 2θ, which agrees with the literature [43]. Relative crystallinity of the samples analyzed was 16.0%, 15.9%, 15.7%, 16.0%, 36.9 and 32.7% for S2, S3, S4, S5, US and Ch, respectively. The mixture of US and Ch to form a film-forming solution decreased the crystallinity of the films, which indicates that the blend was miscible [44]. This behavior agreed with Liu et al. [45] for films prepared using high amylose cornstarch and Ch. The authors attribute the reduction in the film crystallinity to the fact that the hydrogen bonds formed by the interaction between the polmers (US and Ch) can reduce the mobility of the molecules and, therefore, reduce the ability of the components to crystallize.

### 3.8. Fourier-Transform Infrared (ATR-FTIR) Spectroscopy of Starch and Edible Films

**Figure 4:**
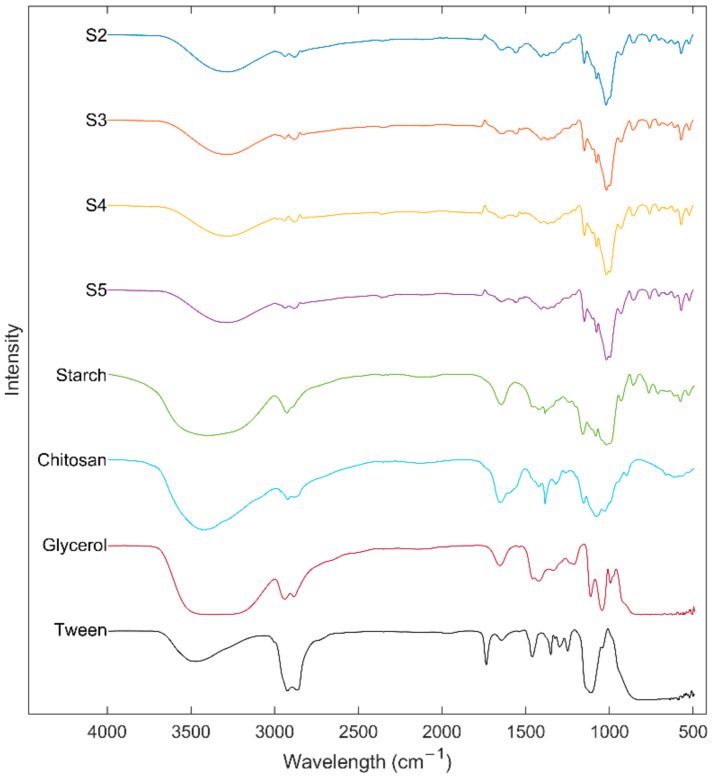
FTIR spectrums of ulluco starch, chitosan, glycerol, tween 80, and ulluco starch–chitosan edible films.

Interaction of the components of edible films was studied using the ATR-FTIR technique. Figure 4 shows the ATR-FTIR spectra of the US, Ch, glycerol, Tween^®^ 80, and edible films. The absorption bands between 3600 and 3000 cm^−1^ observed in pure samples and edible films could be associated with the stretching of free hydroxyl groups [10]. However, this absorption region can also be related to the stretching of amide (–N–H) present in the Ch [20]. Spectra also showed peaks at 2900 cm^−1^ associated with stretching–CH and –CH2 and bending vibrations [46]. The interactions between components of a system are related to changes in the wavenumbers of the spectral peaks [45]. Compared to the ATR-FTIR spectra, a shift of the peaks of hydroxyl groups from US 3386 cm^−1^ and Ch 3427 cm^−1^ to 3288 cm^−1^ in the edible films was observed, which can be related to the formation of inter and intra-molecular hydrogen bonding be0p;.4rfc tween the components [27]. Peaks at 1645 and 1562 cm^−1^ in the edible films indicate hydroxyl-amino group interaction (starch–Ch interaction) and Ch primary amine ionization, respectively [47]. Interaction of the components can be confirmed due to the shift of the peaks at 1656 cm^−1^ of Ch and 1650 cm^−1^ of the starch to 1645 cm^−1^ in the edible films [45]. In addition, it was observed that an increase in starch concentration reduces the intensity of the peaks at 1562 and 1645 cm^−1^. Furthermore, edible films showed peaks at 1089 and 931 cm^−1^ characteristics of the crystalline starch structure and at 1043 cm^−1^ associated with the amorphous region [10].

### 3.9. Thermal Analysis

**Figure 5:**
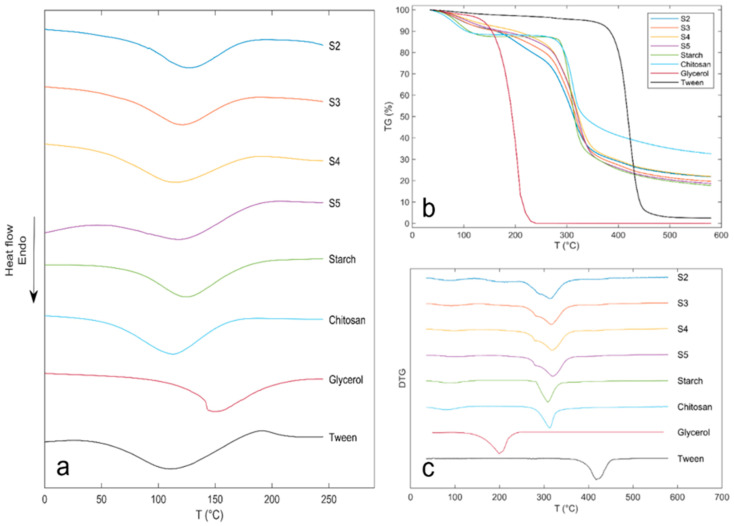
DSC thermogram (**a**), TG (**b**) and DTG (**c**) curves of ulluco starch, chitosan, glycerol, tween 80 and ulluco starch–chitosan edible films.

The DSC thermograms of US–Ch edible films and pure components are shown in Figure 5. Melting points (*T*_m_) of US, Ch, glycerol, and Tween were 128.2, 113.4, 145.2, and 115.4 °C, respectively. As shown in Figure 5a, the endothermic peaks corresponding to *T*_m_ were 119.9, 121.1, 114.5, and 126.3 °C for S2, S3, S4, and S5, respectively (Table 3). Except for S4, the *T*_m_ of the samples showed an increase with the US concentration, which can be associated with the higher concentration of starch, resulting in greater interactions between the hydroxyl and the –NH3 groups of the Ch [45]. This phenomenon can also be explained by MC, since water molecules can act as a plasticizer and weaken the intermolecular forces by hindering the alignment of the polymer chains [48]. Results obtained in this work were similar to those reported for edible films obtained from potato starch and Ch with peanut shell and skin extracts [49].

The thermal decomposition of the films and pure components was evaluated using a TG analysis. Figure 5b,c shows the weight loss and the first derivative of weight loss curves of samples and individual components. Thermal decomposition of film samples took place in three stages. The first stage occurred between 47.7 and 178.9 °C with a weight loss between 8.42% and 10.59%, and the maximum decomposition rate between 87.3 and 99.2 °C (Table 3). This loss of mass is associated with water evaporation inside the film [6]. The second stage of weight loss took place at a temperature between 259.9 and 389.3 °C with a weight loss between 60.74% and 62.57% at the maximum decomposition rate at temperatures between 313.8 and 319.8 °C, which is associated with the depolymerization of starch and Ch molecules and decomposition of functional groups of the Ch, glycerol, and starch, such as hydroxyl and amine groups [10,15]. The third stage occurred between 391.1 and 463.9 °C, with a weight loss between 4.80% and 5.20% and a maximum degradation rate between 418.5 and 419.2 °C; this stage can be associated with the decomposition of the Tween 80, which has a maximum decomposition temperature of 419.5 °C (pure component) (Figure 5c). Decomposition stages one and two showed a shift in the onset and maximum temperatures compared to the pure compounds, probably related to the interaction between Ch/US/water (stage 1) and interactions between Ch/US/Glycerol (stage 2). In addition, an increase in the maximum decomposition temperature of the films was observed, related to an increase in the starch composition, which is due to the increased number of hydroxyl groups. The above improves the interaction between starch functional groups, water molecules, and functional groups of Ch, reinforcing the film network formed. This rise in the onset temperature of film degradation due to the interactions of its components indicates an increase in the stability of the edible films [6,15].

## 4. Conclusions

Edible films based on US and Ch were successfully prepared, indicating a good interaction between the two polymers used. In general, an improvement in mechanical and thermal properties was observed with an increase in US concentration. In contrast, the increase in starch led to a decrease in barrier properties, limiting the number of applications of US-rich packaging. On the other hand, low and medium concentrations of US together with Ch generated films with high barrier capacity. Concerning the visual attributes, all the films evaluated presented low opacity and yellow index, which facilitates their use in products that do not require high protection from light exposure and require the product to be viewed by the consumer. The good thermal properties of the US/Ch films are associated with their potential for use in a wide range of temperatures. The US presents great technological potential as a material for preparing environmentally friendly packaging focused on the food industry. The above can be affirmed due to its high extraction performance, low production costs, high amylose content, and ease of interaction with other polymers such as Ch. However, more studies are needed to better understand the US interaction with other polymers and bioactive compounds.

## Figures and Tables

**Table 1:** Ulluco starch /Chitosan edible film formulations.

Sample	Initial Concentration of Solutions		Final Concentration of Films			
	US (%)	Ch (%)	US (%)	Ch (%)	Glycerol (%)	Tween 80^®^ (%)
S2	2.0	1.5	1.0	0.75	1	0.1
S3	3.0	1.5	1.5	0.75	1	0.1
S4	4.0	1.5	2.0	0.75	1	0.1
S5	5.0	1.5	2.5	0.75	1	0.1

US: ulluco starch; Ch: chitosan.

**Table 2:** Physicochemical properties of ulluco starch–chitosan edible films.

Sample	MC (%)	Solubility (%)	SP (%)	WVP × 10^−11^ (g/m s Pa)	Opacity	YI
S2	33.4 ± 1.2 ^a^	17.5 ± 1.0 ^c^	39 ± 5 ^d^	5.6 ± 0.3 ^c^	0.40 ± 0.06 ^a^	5.7 ± 1.7 ^a^
S3	21.1 ± 1.6 ^b^	19.6 ± 0.7 ^b^	116 ± 20 ^c^	6.9 ± 0.5 ^b^	0.32 ± 0.02 ^b^	5.3 ± 0.9 ^a^
S4	17.9 ± 1.2 ^c^	19.7 ± 0.8 ^b^	177 ± 22 ^b^	6.4 ± 0.7 ^bc^	0.32 ± 0.03 ^b^	4.1 ± 0.4 ^b^
S5	15.2 ± 0.8 ^d^	21.7 ± 1.1 ^a^	267 ± 18 ^a^	8.5 ± 0.9 ^a^	0.31 ± 0.01 ^b^	4.1 ± 0.4 ^b^

MC: moisture content; S: solubility; SP: swelling power; WVP: water vapor permeability; WI: white index; YI: yellow index; Values are expressed as means ± standard deviation. Different letters in the same column indicate significant differences between samples (*p* ≤ 0.05).

**Table 3:** Thermal degradation temperature of ulluco starch–chitosan edible films and pure components.

Sample	*T*_m_ (°C)	Thermal Degradation Temperature (°C)						Total Loss (%)
		Peak 1		Peak 2		Peak 3		
		*T*_onset_ (°C)	*T*_max_ (°C)	*T*_onset_ (°C)	*T*_max._ (°C)	*T*_onset_ (°C)	*T*_max._ (°C)	
S2	119 ± 9.52	47.7	87.3	259.9	313.8	398.6	418.5	79.7
S3	121 ± 0.98	47.7	91.9	263.9	316.5	391.1	419.2	80.3
S4	114 ± 4.16	52.1	99.2	261.9	318.6	396.5	418.5	78.1
S5	126 ± 5.60	52.1	98.4	263.9	319.8	393.5	418.4	81.5
Glycerol	145 ± 1.99	60.2	103.9	230.7	273.4	-	-	97.5
Tween 80^®^	115 ± 9.34	392.4	419.5	-	-	-	-	100
Starch	128 ± 5.26	50.0	89.3	281.9	308.5	-	-	78.3
Chitosan	113 ± 0.66	45.7	82.6	282.4	312.2	-	-	67.7

## Data Availability

Data is contained within the article.

## References

[1] Plastics Europe The Facts 2016: An Analysis of European Plastics Production, Demand and Waste Data 2016. https://plasticseurope.org/wp-content/uploads/2021/10/2016-Plastic-the-facts.pdf.

[2] Shapi’I R.A., Othman S.H., Nordin N., Basha R.K., Naim M.N. (2020). Antimicrobial properties of starch films incorporated with chitosan nanoparticles: In vitro and in vivo evaluation. Carbohydr. Polym..

[3] Spranz R., Schlüter A., Vollan B. (2018). Morals, money or the master: The adoption of eco-friendly reusable bags. Mar. Policy.

[4] Nielsen T.D., Holmberg K., Stripple J. (2019). Need a bag? A review of public policies on plastic carrier bags–Where, how and to what effect?. Waste Manag..

[5] Silva V.D.M., Macedo M.C.C., Rodrigues C.G., dos Santos A.N., Loyola A.C.D.F.E., Fante C.A. (2020). Biodegradable edible films of ripe banana peel and starch enriched with extract of *Eriobotrya japonica* leaves. Food Biosci..

[6] Zheng K., Xiao S., Li W., Wang W., Chen H., Yang F., Qin C. (2019). Chitosan-acorn starch-eugenol edible film: Physico-chemical, barrier, antimicrobial, antioxidant and structural properties. Int. J. Biol. Macromol..

[7] Towle M.A. (1961). The Ethnobotany of Pre-Columbian Peru.

[8] Cruz G., Ribotta P., Ferrero C., Iturriaga L. (2016). Physicochemical and rheological characterization of Andean tuber starches: Potato (*Solanum tuberosumssp.* Andigenum), Oca (*Oxalis tuberosa* Molina) and Papalisa (*Ullucus tuberosus* Caldas). Starch-Stärke.

[9] Valcárcel-Yamani B., Rondan-Sanabria G.G., Finardi-Filho F. (2013). The physical, chemical and functional characterization of starches from Andean tubers: Oca (*Oxalis tuberosa* Molina), olluco (*Ullucus tuberosus* Caldas) and mashua (*Tropaeolum tuberosum* Ruiz & Pavón). Braz. J. Pharm. Sci..

[10] Galindez A., Daza L.D., Homez-Jara A., Eim V.S., Váquiro H.A. (2019). Characterization of ulluco starch and its potential for use in edible films prepared at low drying temperature. Carbohydr. Polym..

[11] Daza L.D., Homez A., Solanilla J.F., Váquiro H.A. (2018). Effects of temperature, starch concentration, and plasticizer concentration on the physical properties of ulluco (*Ullucus tuberosus* Caldas)-based edible films. Int. J. Biol. Macromol..

[12] Wang B., Sui J., Yu B., Yuan C., Guo L., El-Aty A.A., Cui B. (2020). Physicochemical properties and antibacterial activity of corn starch-based films incorporated with Zanthoxylum bungeanum essential oil. Carbohydr. Polym..

[13] Podshivalov A., Zakharova M., Glazacheva E., Uspenskaya M. (2017). Gelatin/potato starch edible biocomposite films: Correlation between morphology and physical properties. Carbohydr. Polym..

[14] Sandhu K.S., Sharma L., Kaur M., Kaur R. (2020). Physical, structural and thermal properties of composite edible films prepared from pearl millet starch and carrageenan gum: Process optimization using response surface methodology. Int. J. Biol. Macromol..

[15] Hasan M., Rusman R., Khaldun I., Ardana L., Mudatsir M., Fansuri H. (2020). Active edible sugar palm starch-chitosan films carrying extra virgin olive oil: Barrier, thermo-mechanical, antioxidant, and antimicrobial properties. Int. J. Biol. Macromol..

[16] Khan F., Pham D.T.N., Oloketuyi S.F., Manivasagan P., Oh J., Kim Y.-M. (2020). Chitosan and their derivatives: Antibiofilm drugs against pathogenic bacteria. Colloids Surf. B Biointerfaces.

[17] Pacheco A.C.S., Sousa F., Sarmento B. (2020). Chitosan-based nanomedicine for brain delivery: Where are we heading?. React. Funct. Polym..

[18] El-hack M.E.A., El-saadony M.T., Sha M.E., Zabermawi N.M., Arif M., Elsaber G., Khafaga A.F., El-hakim Y.M.A., Al-sagheer A.A. (2020). Antimicrobial and antioxidant properties of chitosan and its derivatives and their applications: A review. Int. J. Biol. Macromol..

[19] Luchese C.L., Pavoni J.M.F., Dos Santos N.Z., Quines L.K., Pollo L.D., Spada J.C., Tessaro I.C. (2018). Effect of chitosan addition on the properties of films prepared with corn and cassava starches. J. Food Sci. Technol..

[20] Homez-Jara A., Daza L.D., Aguirre D.M., Muñoz J.A., Solanilla J.F., Váquiro H.A. (2018). Characterization of chitosan edible films obtained with various polymer concentrations and drying temperatures. Int. J. Biol. Macromol..

[21] ASTM (2016). Standard Test Methods for Water Vapor Transmission of Materials.

[22] ASTM (2018). Standard Test Method for Tensile Properties of Thin Plastic Sheeting.

[23] Mohajer S., Rezaei M., Hosseini S.F. (2017). Physico-chemical and microstructural properties of fish gelatin/agar bio-based blend films. Carbohydr. Polym..

[24] Khedri S., Sadeghi E., Rouhi M., Delshadian Z., Mortazavian A.M., Guimarães J.D.T., Fallah M., Mohammadi R. (2021). Bioactive edible films: Development and characterization of gelatin edible films incorporated with casein phosphopeptides. LWT.

[25] Jha P. (2020). Effect of plasticizer and antimicrobial agents on functional properties of bionanocomposite films based on corn starch-chitosan for food packaging applications. Int. J. Biol. Macromol..

[26] Thakur R., Saberi B., Pristijono P., Stathopoulus C.E., Golding J.B., Scarlett C.J., Bowyer M., Vuong Q.V. (2017). Use of response surface methodology (RSM) to optimize pea starch-chitosan novel edible film formulation. J. Food Sci. Technol..

[27] Ren L., Yan X., Zhou J., Tong J., Su X. (2017). Influence of chitosan concentration on mechanical and barrier properties of corn starch/chitosan films. Int. J. Biol. Macromol..

[28] Pająk P., Przetaczek-Rożnowska I., Juszczak L. (2019). Development and physicochemical, thermal and mechanical properties of edible films based on pumpkin, lentil and quinoa starches. Int. J. Biol. Macromol..

[29] Mathew S., Brahmakumar M., Abraham T.E. (2006). Microstructural imaging and characterization of the mechanical, chemical, thermal, and swelling properties of starch–chitosan blend films. Biopolymers.

[30] Vásconez M.B., Flores S., Campos C.A., Alvarado J., Gerschenson L.N. (2009). Antimicrobial activity and physical properties of chitosan–tapioca starch based edible films and coatings. Food Res. Int..

[31] Bof M.J., Bordagaray V.C., Locaso D.E., García M.A. (2015). Chitosan molecular weight effect on starch-composite film properties. Food Hydrocoll..

[32] Dang K.M., Yoksan R. (2016). Morphological characteristics and barrier properties of thermoplastic starch/chitosan blown film. Carbohydr. Polym..

[33] Basiak E., Lenart A., Debeaufort F. (2017). Effect of starch type on the physico-chemical properties of edible films. Int. J. Biol. Macromol..

[34] Vogler E.A. (1998). Structure and reactivity of water at biomaterial surfaces. Adv. Colloid Interface Sci..

[35] Marvdashti L.M., Koocheki A., Yavarmanesh M. (2017). Alyssum homolocarpum seed gum-polyvinyl alcohol biodegradable composite film: Physicochemical, mechanical, thermal and barrier properties. Carbohydr. Polym..

[36] Jayasekara R., Harding I., Bowater I., Christie G., Lonergan G. (2004). Preparation, surface modification and characterisation of solution cast starch PVA blended films. Polym. Test..

[37] Filho J.G.D.O., Bezerra C.C.D.O.N., Albiero B.R., Oldoni F.C.A., Miranda M., Egea M.B., de Azeredo H.M.C., Ferreira M.D. (2020). New approach in the development of edible films: The use of carnauba wax micro- or nanoemulsions in arrowroot starch-based films. Food Packag. Shelf Life.

[38] Zhang S., Kim N., Yokoyama W., Kim Y. (2018). Effects of moisture content on mechanical properties, transparency, and thermal stability of yuba film. Food Chem..

[39] Pereda M., Ponce A.G., Marcovich N.E., Ruseckaite R.A., Martucci J.F. (2011). Chitosan-gelatin composites and bi-layer films with potential antimicrobial activity. Food Hydrocoll..

[40] Chillo S., Flores S., Mastromatteo M., Conte A., Gerschenson L., Del Nobile M. (2008). Influence of glycerol and chitosan on tapioca starch-based edible film properties. J. Food Eng..

[41] Talón E., Trifkovic K.T., Nedovic V.A., Bugarski B.M., Vargas M., Chiralt A., González-Martínez C. (2017). Antioxidant edible films based on chitosan and starch containing polyphenols from thyme extracts. Carbohydr. Polym..

[42] Silva-Weiss A., Bifani V., Ihl M., Sobral P., Gómez-Guillén M. (2014). Polyphenol-rich extract from murta leaves on rheological properties of film-forming solutions based on different hydrocolloid blends. J. Food Eng..

[43] Wang L., Ding J., Fang Y., Pan X., Fan F., Li P., Hu Q. (2020). Effect of ultrasonic power on properties of edible composite films based on rice protein hydrolysates and chitosan. Ultrason. Sonochem..

[44] Yusof Y.M., Shukur M.F., Illias H.A., Kadir M.F.Z. (2014). Conductivity and electrical properties of corn starch–chitosan blend biopolymer electrolyte incorporated with ammonium iodide. Phys. Scr..

[45] Liu H., Adhikari R., Guo Q., Adhikari B. (2012). Preparation and characterization of glycerol plasticized (high-amylose) starch–chitosan films. J. Food Eng..

[46] Gahruie H.H., Mostaghimi M., Ghiasi F., Tavakoli S., Naseri M., Hosseini S.M.H. (2020). The effects of fatty acids chain length on the techno-functional properties of basil seed gum-based edible films. Int. J. Biol. Macromol..

[47] Chakravartula S.S.N., Lourenço R.V., Balestra F., Bittante A.M.Q.B., Sobral P.J.D.A., Rosa M.D. (2020). Influence of pitanga (*Eugenia uniflora* L.) leaf extract and/or natamycin on properties of cassava starch/chitosan active films. Food Packag. Shelf Life.

[48] Jaramillo C.M., Gutiérrez T.J., Goyanes S., Bernal C., Famá L. (2016). Biodegradability and plasticizing effect of yerba mate extract on cassava starch edible films. Carbohydr. Polym..

[49] Meng W., Shi J., Zhang X., Lian H., Wang Q., Peng Y. (2020). Effects of peanut shell and skin extracts on the antioxidant ability, physical and structure properties of starch-chitosan active packaging films. Int. J. Biol. Macromol..

